# Light-sheet laser speckle imaging for cilia motility assessment

**DOI:** 10.1016/j.csbj.2023.02.036

**Published:** 2023-02-20

**Authors:** Kai Long, Jing Liu, Shuhao Shen, Mark Thong, Deyun Wang, Nanguang Chen

**Affiliations:** a Department of Biomedical Engineering, National University of Singapore, 4 Engineering Drive 3, S117583, Singapore; b Department of Otolaryngology, Infectious Diseases Translational Research Program, Yong Loo Lin School of Medicine, National University of Singapore, National University Health System, Singapore 119228, Singapore; c Xidian University, Guangzhou Institute of Technology, Guangzhou 510555, China; d Department of Otolaryngology-Head and Neck Surgery, National University Hospital, National University Health System, Singapore; e NUS (Suzhou) Research Institute, No. 377 Linquan Street, Suzhou Industrial Park, Suzhou, Jiangsu, China

**Keywords:** Cilia, Motility, Laser speckle imaging, Autocorrelation, PIV

## Abstract

Mucociliary clearance is an important innate defense mechanism predominantly mediated by ciliated cells in the upper respiratory tract. Ciliary motility on the respiratory epithelium surface and mucus pathogen trapping assist in maintaining healthy airways. Optical imaging methods have been used to obtain several indicators for assessing ciliary movement. Light-sheet laser speckle imaging (LSH-LSI) is a label-free and non-invasive optical technique for three-dimensional and quantitative mapping of velocities of microscopic scatterers. Here, we propose to use an inverted LSH-LSI platform to study cilia motility. We have experimentally confirmed that LSH-LSI can reliably measure the ciliary beating frequency and has the potential to provide many additional quantitative indicators for characterizing the ciliary beating pattern without labeling. For example, the asymmetry between the power stroke and the recovery stroke is apparent in the local velocity waveform. PIV (particle imaging velocimetry) analysis of laser speckle data could determine the cilia motion directions in different phases.

## Graphical Abstract

**Figure:**
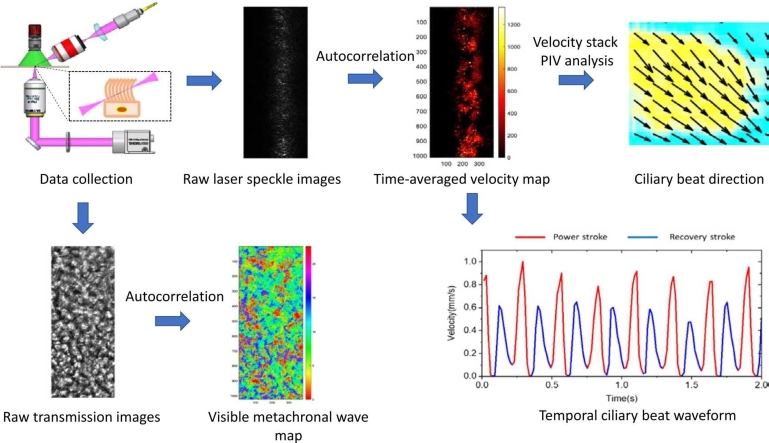

## 1 Introduction

The human airway mucosa is lined by a pseudostratified epithelium that acts as the first line of defense against inhaled infectious pathogens and physical insults. Ciliated cells are the predominant epithelial cells (over 50%, possessing 200–300 cilia per cell) in the upper respiratory tract and beat in a coordinated manner with a regular frequency and wave pattern [1], [2]. It provides normal clearance of mucus and debris from the upper airways and is essential to maintaining healthy airway mucosa. Under certain circumstances, the defects of cilia and susceptibility of cilia to exogenous and endogenous stimuli might induce impaired ciliary function [3], [4], [5], [6]. The impaired ciliary function is a common pathological feature in patients with chronic airway diseases, resulting in worsening airway obstruction and increased susceptibility to respiratory infections[7]. Hence, visualization and detection of ciliary function provide valuable information for disease diagnosis.

At present, the functional dynamics of beating cilia are evaluated based on the ciliary beat frequency (CBF) and/or ciliary beat pattern (CBP). Studies conducted on airway ciliary cells have shown that the magnitude of CBF is an important indicator of the ciliary transport process[8], [9]. The CBP is a collection of measures that are related to the geometric and kinetic information of the cilia under investigation. Previous studies have shown that various stimulations changed the CBP in addition to the CBF [10], [11], [12]. The CBP analysis seeks to evaluate ciliary characteristics such as direction, coordination, and stiffness, all of which have the potential to have an effect on ciliary effectiveness. For instance, normal CBPs are often characterized by distinguishable regular forward and recovery strokes, whereas abnormal CBPs might be characterized by static cilia with limited motions, uncoordinated beating, or irregular circular beating. As certain aberrant patterns may impair mucus movement without a low CBF. Thus, these descriptions of ciliary motion are more informative than CBF alone and CBP analysis may reveal the nature of the cilia defect. When combined with CBF and CBP testing, the sensitivity and specificity of the procedure for identifying aberrant ciliary motility are significantly improved.

Our knowledge of ciliary motility in healthy and diseased conditions has been significantly expanded as a result of recent developments in imaging technologies. Diagnostics might be improved by the use of imaging modalities such as wide-field transmission microscopy, differential interference contrast (DIC) microscopy, epifluorescence microscopy, high-speed video microscopy, and optical coherence tomography and they could also be applied for precision medicine.[13], [14], [15].

In the conventional evaluation setup, the cilia sample is probed by a light beam that passes through the sample perpendicularly [16], [17], [18], [19], [20]. The transmitted light is subject to time-dependent intensity modulation by the beating cilia. A single photodetector such as a photodiode or a photosensitive cell is typically used to pick up the undulating light signal directly from the sample or indirectly from a monitor. After Fourier domain analysis of the photoelectrical signal, it is possible to identify the main peak and the corresponding CBF. Although it is effective for visualizing single cilia motions, it requires adequate imaging speed to acquire quantitative CBF measurements and record the whole beating cycle. Moreover, the single photodetector approach is prone to environmental vibration and cannot be used to evaluate a large field of view and also does not allow for the evaluation of the CBP. More recently, high-speed cameras became available and affordable. They have been integrated into microscopy platforms to capture high-frame-rate, high-magnification video sequences. Using microscopy with a high-speed camera (500 Hz), it is feasible to analyze the ciliary beat cycles, beat pattern, amplitude, degree, and speed which are then processed to derive the CBF and CBP [20], [21], [22]. The automation of CBF calculations has been mostly successful, but a valid measure of CBP still needs to be done by manually[23], [24]. Nonetheless, a standard procedure for CBF and CBP analysis by high-speed video microscopy is not yet sufficiently established. [25], [26]Nevertheless, these microscopic platforms are essentially based on the detection of the transmitted light through the samples. They do not have the optical sectioning capability and depth resolution. In addition, the contrast tends to be low as the transparent sample typically introduces weak perturbations in the transmitted light intensity. The limited imaging performance is a bottleneck for assessing the motile cilia more quantitatively. To circumvent these constraints, optical coherence tomography (OCT) has recently been used to observe ciliary motion by using its micron-scale resolution, real-time imaging capability, and endoscopic imaging potential[27], [28], [29]. A recent study used a phase-resolved Doppler OCT (PRD-OCT) technique to probe the beating direction and speed of cilia to further expand the effectiveness of using an OCT system in imaging ciliary activity[29]. However, the complete cilia structure cannot yet be measured over a three-dimensional plane by OCT. The capacity to identify anomalies in CBP would be considerably improved by three-dimensional imaging at the resolution offered by OCT.

Laser speckle imaging (LSI) is an established label-free imaging technique for flow visualization. The LSI system illuminates a sample with a coherent laser beam, which is scattered by randomly distributed microscopic scatterers inside the sample. The scattered waves from individual scatterers interfere with each other and generate a random, alternating bright and dark pattern, the so-called laser speckle [30]. When the microscopic scatterers start to move, the speckle pattern will change dynamically. The LSI system collects the speckle patterns and analyses their temporal and/or spatial statistic behaviors to retrieve information about the motion [31]. LSI has found many applications in visualizing retinal perfusion, cerebral blood flow, and skin microvasculature [32], [33], [34], [35], [36].

In our previous studies, we have developed novel LSI platforms to further improve imaging performance. A line scan laser speckle autocorrelation imaging (LS-LSAI) method was proposed to address the two major drawbacks of conventional laser speckle imaging techniques, namely the poor quantification accuracy and limited penetration [37], [38]. Recently, we reported a light-sheet laser speckle imaging (LSH-LSI) system [39]. Zebrafish imaging experiments with LSH-LSI have demonstrated its excellent three-dimensional resolutions and velocity quantification accuracy. Due to its ultrahigh imaging speed, LSH-LSI is also capable of vectorial flow map generation. In this study, we built and optimally configured an inverted LSH-LSI system for visualizing and quantifying the motion of motile cilia. We conducted imaging experiments with the air-liquid interface (ALI) cultured nasal epithelial cells. It has been verified that this new method can measure the CBF and its changes in response to a variety of stimulations. Many potential CBP indicators could be obtained from quantitatively measured local velocity waveforms and motion directional changes in different beating phases.

## 2 Materials & methods

### 2.1 Setup

**Fig. 1:**
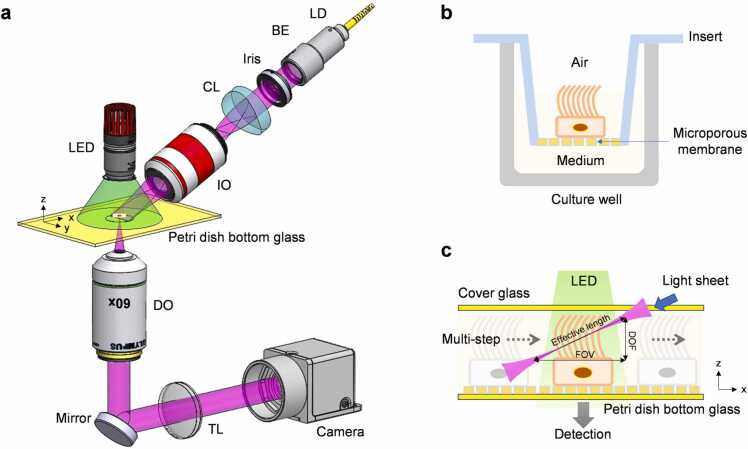
Schematic diagram of inverted light-sheet laser speckle imaging system. (a) The inverted LHS-LSI system contained three parts, illumination light path (above the yellow Petri dish plane), a sample stage, and detection light path (below the yellow Petri dish plane). The illumination light path had two light sources. One was the laser source for detecting laser speckle images, and the other was LED for detecting transmission images. LD, laser diode; BE, beam expander; CL, cylindrical lens; IO, illumination objective; DO, detection objective; TL, tube lens. (b) Air-liquid interface (ALI) culture system. The ciliary cells were cultivated on the microporous membrane. The lower surface of the cell cluster contacted the medium through the microporous, and the upper surface of the cell cluster was exposed to the air directly. (c) The sample was fixed between the cover glass and the Petri dish bottom glass. The laser light sheet entered the sample obliquely from the top-right, and the LED illuminated the sample from the top. The position of the sample was shifted by actuating the sample stage.

Fig. 1**(a)** shows the schematic diagram of the inverted light-sheet laser speckle imaging system. The sample carried by a Petri dish was fixed on the sample stage (not shown in Fig. 1), while the illumination beam entered the sample from the top surface and the detection beam propagated downwards. The output from a 405 nm laser diode was collimated, and then the parallel beam was expanded by a beam expander (5 ×, Edmund Optics). A cylindrical lens (LJ1695RM-A, focal length 50 mm, Thorlabs) was used to condense the expanded parallel beam and generate a light sheet after the illumination objective lens (123TL/05, 4 ×/0.1NA, Leica). The effective thickness and length of the light sheet in the focal region were controlled by an iris aperture between the beam expander and the cylindrical lens. By removing the cylindrical lens, we could quickly switch to conventional surface illumination as an alternative. Vertically above the sample, a LED (central wavelength 520 nm) provided the wide-field illumination for acquiring transmission images, which could be used as complementary information or for making cross-validation. Along the detection axis, the scattered photons were collected by a high-magnification, water immersed detection objective (LUMPLFLN60XW, 60 ×/1.0NA, working distance 2 mm, Olympus) and a tube lens (f=200 mm). The speckle patterns within the depth of focus (DOF) were projected to the sensing area of a high-speed camera (pco.dimax cs1, Excelitas Technologies® Corp, Germany) whose full-frame (1296 ×1024 pixels, pixel size 11 ×11 µm^2^) rate can reach up to 3086 frames per second (fps). The speckle images and transmission images were usually collected in sequence by turning only the laser or LED on at a time.

The microporous membrane with ciliary cells was torn off from the insert with a tweezer (as shown in Fig. 1**(b)**), and affixed to the bottom glass of the petri dish (as shown in Fig. 1**(c)**). Different from in vivo blood perfusion measurements, the motile cilia for in vitro experiments needed to be immersed in the medium to keep the sample active. The medium fully filled the gap between the Petri dish bottom glass and the cover glass. To acquire laser speckle images slice by slice, the sample could be shifted with a specific step size along the x-axis, as indicated by the dashed arrows in Fig. 1**(c)**. The sample stage was driven by an actuator (DMX3104SH-01, NTN), which was controlled by a DAQ (data acquisition) device (USB6008, National Instruments, USA).

### 2.2 Data processing

Raw laser speckle images can be analyzed with temporal autocorrelation to find the decorrelation time and the corresponding velocity. According to established mathematical model [40], the laser speckle intensity autocorrelation function $g_{2}\left(\tau \right)$ and the intensity decorrelation time $\tau_{c}$ can be related directly as

(1) $$g_{2}\left(\tau \right)=1+{\beta \left[e^{-{\left(\tau /\tau_{c}\right)}^{n}}\right]}^{2},$$

where β is the correction factor associated with the illumination geometry and n depends on the light scattering regime and motion types of the light scatterers. As the scattered light from the transparent sample was dominated by single scattering, we chose n=1 in this study. The movement velocity can be converted from the decorrelation time $\tau_{c}$ using the formula

(2) $$v=\frac{\lambda }{\pi *\mathrm{NA}{*\tau }_{c}},$$

where λ is the laser source wavelength, and NA is the numerical aperture of the detection (imaging) objective. Autocorrelation was performed on the image intensity pixel by pixel, with a moving time window [41] that typically covered 20 frames.

Particle Image Velocimetry (PIV) is a velocity measurement technique, which calculates a displacement vector for each interrogation window in the FOV with the aid of autocorrelation or cross-correlation techniques. PIVlab is a user-friendly, graphical user interface (GUI) based digital particle image velocimetry software embedded in Matlab [42]. In this study, we applied PIV analysis on raw speckle images to identify the local moving direction of scatterers. More specifically, we used PIVlab to generate vector velocity maps.

### 2.3 Sample preparation

The human nasal epithelial cells (hNECs) were differentiated from human nasal epithelial stem/progenitor cells isolated from the inferior turbinate of healthy subjects who underwent septal plastic surgery at the National University Hospital of Singapore. Approval to conduct this study was obtained from the National Healthcare Group Domain-Specific Board of Singapore (DSRB code D/11/228). The hNECs were transferred to an ALI culture system to differentiate into ciliated cells within 4 weeks. As illustrated in Fig. 1**(b)**, the ciliated cells were cultivated on the microporous membrane of a transwell insert, the size of the microporous is 0.4 µm, and the diameter of the membrane is 6.5 mm. Methods for culturing hNECs were described in the previous paper [43]. According to previous studies [11], [12], we used 10 ng/ml Interleukin-13 (IL-13 R&D System, Minneapolis, MN, USA) and 50 μM of Zinc chloride (ZnCl_2,_ Sigma, USA) solution to induce the reduction and enhancement of cilia beating.

## 3 Results

### 3.1 Oblique light-sheet laser speckle imaging system characteristics

Light-sheet microscopy has the intrinsic optical sectioning capability, in which the illumination beam and the detection beam are typically orthogonal. For laser speckle autocorrelation imaging, the exposure time needs to be as short as possible to capture the instant speckle pattern. In addition, published studies have shown that the reciprocating frequency of airway epithelia ciliary cells is 15–25 Hz [44], which demands an adequately high frame rate to satisfy the Nyquist sampling law. For the above two reasons, the system was configured in a way such that the illumination beam and the detection beam formed a large angle, which was greater than 90° (see in Fig. 1**(a)** and 1**(c)**). The oblique light sheet enabled the detection of forward-scattering photons, which were much stronger than backward-scattering photons, resulting in better imaging quality and faster image acquisition [39]. The inverted optical system implementation with a water immersed detection objective helped minimize the impact of the environmental disturbance on the air-liquid interface. It also simplified the sample preparation and improved measurement efficiency. In this study, the incident illumination light formed a 20° angle with the horizontal Petri dish. After refraction at the air-water interface, the light sheet was tilted at 45° with respect to the detection optical axis. Consequently, the scattering angle was approximately 135°. The effective length of the light sheet was around 54 µm when the iris was fully open and could be extended for a reduced iris diameter. The width of the light sheet was typically a few millimeters and therefore was not a limiting factor in image acquisition.

### 3.2 Light-sheet vs. surface illumination

Surface illumination is the conventional way to perform laser speckle imaging. We have conducted a comparison study to demonstrate the advantages to use light sheet illumination for better quantification of motile cilia. Both LSH-LSI and surface illumination LSI (SI-LSI) raw images were acquired at 3000 frames per second (fps) for 1 s for the same FOV (383 × 1012 pixels) from the same sample. After image processing, 300 frames of velocity maps were generated from each raw image stack.

**Fig. 2:**
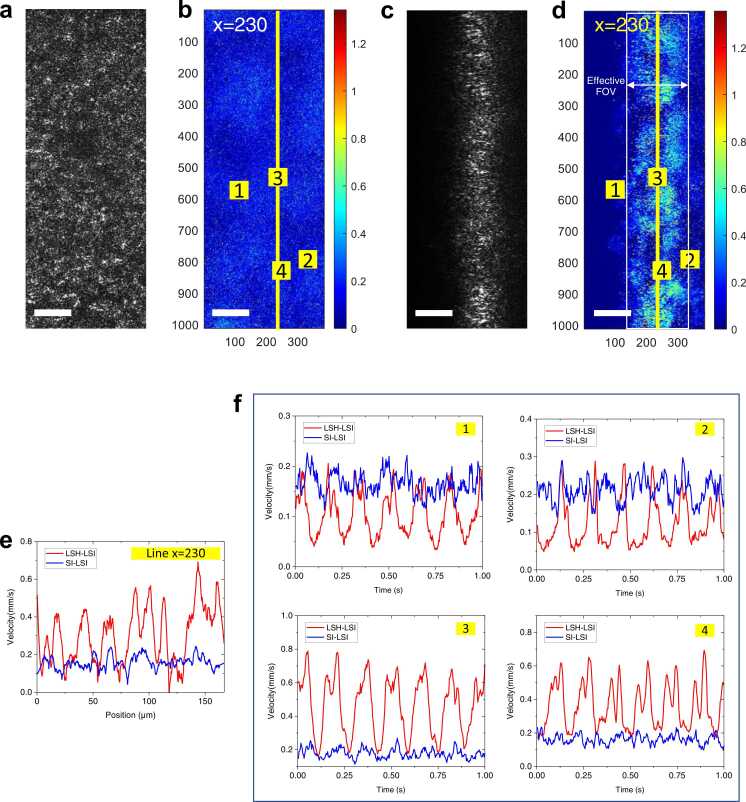
Comparison of LSH-LSI and SI-LSI in the same field of view. **(a)** Raw SI-LSI image. The overall FOV is 66.9 µm × 176.7 µm. **(b)** Time-averaged velocity map of SI-LSI. The axes are labeled in pixels. **(c)** Raw LSH-LSI image. **(d)** Time-averaged velocity map of LSH-LSI. The axes in labeled in pixels. The effective FOV is 24.2 µm × 176.7 µm. **(e)** Comparison of SI-LSI and LSH-LSI velocities along yellow lines in **(b)** and **(d)**. **(f)** Velocity waveform comparison between LSH-LSI and SI-LSI in Regions 1, 2, 3, and 4. Scale bars: 20 µm. **(d)** and **(f)** have the same color bar and the unit is mm/s.

Fig. 2**(a)** and Fig. 2**(c)** are examples of raw images obtained with SI-LSI and LSH-LSI, respectively. Fig. 2**(b)** and Fig. 2**(d)** show corresponding velocity maps time averaged over 1 s. The effective FOV is marked in Fig. 2**(d)** by a white box, which was approximately 24.2 µm in length (indicated by the arrows). The yellow lines in Fig. 2**(b)** and **(d)** were the central position (x = 230) of the light sheet. The axes of these two figures were labeled in pixels. The time-averaged velocity profiles along the yellow lines in Fig. 2**(b)** and Fig. 2**(d)** were compared in Fig. 2**(e)**. We found that light sheet illumination afforded better contrast as well as visually higher spatial resolution than surface illumination. Multiple regions (each region is 40 × 40 pixels) were selected to compare the dynamic characteristics between SI-LSI and LSH-LSI. The instantaneous velocities were spatially averaged in small areas marked by yellow squares (Region 1, 2, 3 & 4) in both Fig. 2**(b)** and **(d)**. Then spatially averaged velocities as a function of time were plotted in Fig. 2**(f)**. The dynamic changes in the cilia moving speed could be better captured by LSH-LSI with improved signal visibility. As shown in Fig. 2**(d)**, Regions 1 and 2 were at the edges of the effective FOV while Regions 3 and 4 were near the center line. Comparing LSH-LSI velocity waveforms in these four regions, we found that peak values in Region 3 and 4 were larger than that in Region 1 and 2. The differences were due to reduced signal-to-noise ratio at the effective FOV edges and the consequently compromised accuracy in velocity estimation. More importantly, it was observed that SI-LSI was much inferior to LSH-LSI in terms of capturing dynamic, periodic changes in velocities. We believe that the inadequacy in SI-LSI technical performance was largely attributed to the lack of optical sectioning, which was the main difference between SI-LSI and LSH-LSI. The scattered speckle patterns originated from different layers were superimposed on each other, leading to speckle averaging and therefore reduced intensity fluctuations in both time and spatial domains. Consequently, the simple mathematic model (Eq. (1)) led to measurement errors.

### 3.3 Ciliary beat frequency

**Fig. 3:**
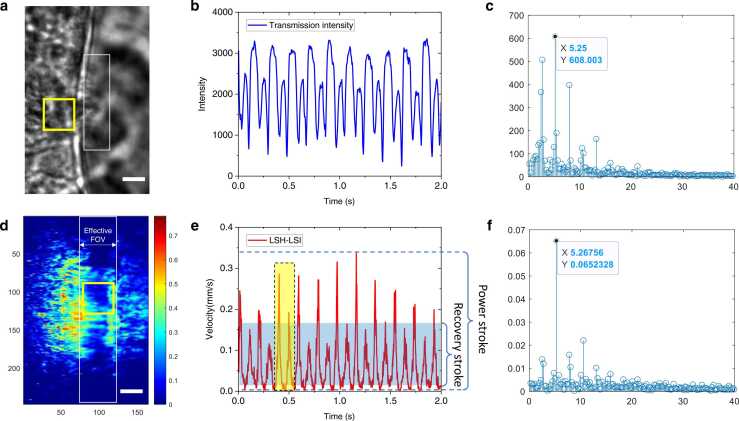
Cross-validation for ciliary beat frequency. **(a)** Transmission image of the ciliary cell. The FOV is 28.6 µm × 43.1 µm. **(b)** Transmission intensity waveform spatially averaged over the yellow box in **(a)**. **(c)** Fourier-transform of the transmission intensity waveform of **(b)**. **(d)** Time-averaged velocity map over 2 s. The unit of the color bar is mm/s. **(e)** Velocity waveform spatially averaged over the yellow box in **(d)**. **(f)** Fourier-transform of the velocity waveform in **(e)**. Scalar bars: 5 µm.

The ciliary beat frequency (CBF) is the paramount indicator to measure the health of the airway. Cross-validation was applied to highlight the capability of LSH-LSI in evaluating the CBF by comparing it with transmission imaging. The transmission images, as shown in Fig. 3**(a)**, were collected at 300 fps for 2 s, and subsequently, the light-sheet speckle images were captured at 3000 fps also for 2 s. The spatially averaged transmission image intensity in the yellow box (40 × 40 pixels) marked in Fig. 3**(a)** as a function of time was plotted in Fig. 3**(b)**. There were alternating high peaks and low peaks that repeated themselves after regular time intervals. Each high-low peak pair constituted one complete ciliary beat, as indicated by the yellow square in Fig. 3**(e)**. From the intensity waveform, the period of the ciliary beat was estimated to be 0.188 ± 0.004 s. The frequency analysis resulted in a ciliary beat frequency of about 5.25 Hz, as shown in Fig. 3**(c)**. Fig. 3**(d)** is the velocity map time-averaged over 600 frames in 2 s. The instantaneous velocities spatially averaged over interrogation regions (indicated by yellow boxes in Fig. 3**(d)** were plotted in Fig. 3**(e)**. Similar to the transmission imaging results, the high-low peak pairs were also visually identifiable in Fig. 3**(e)**. The beating period was 0.191 ± 0.007 s by finding the average time interval between consecutive high peaks. The frequency of the ciliary beat obtained from Fourier analysis was 5.26 Hz (**see** Fig. 3**(f)**), which agreed very well with the transmission imaging results.

The above comparison demonstrated that LSH-LSI was capable of accurately measuring the CBF. Moreover, the spatiotemporally varying velocities measured with LSH-LSI could potentially lead to more quantitative indicators. In the velocity waveforms, the higher peaks were associated with the power strokes of cilia while the lower peaks were related to the recovery strokes. These peak values could be compared across samples and/or for the same sample in different environments. In addition, the full width at half maximum (FWHM) of the spikes in the velocity waveform peaks could be used to quantify the durations of the power stroke and recovery stroke. In this study, the FWHM of the power stroke duration was 0.023 ± 0.003 s, and the FWHM of the recovery stroke was 0.035 ± 0.004 s. The power stroke velocity is 0.25 ± 0.04 mm/s, and the recovery stroke velocity is 0.15 ± 0.02 mm/s. The high-to-low peak ratio was 1.74.

### 3.4 Ciliary beat frequency modulation

**Fig. 4:**
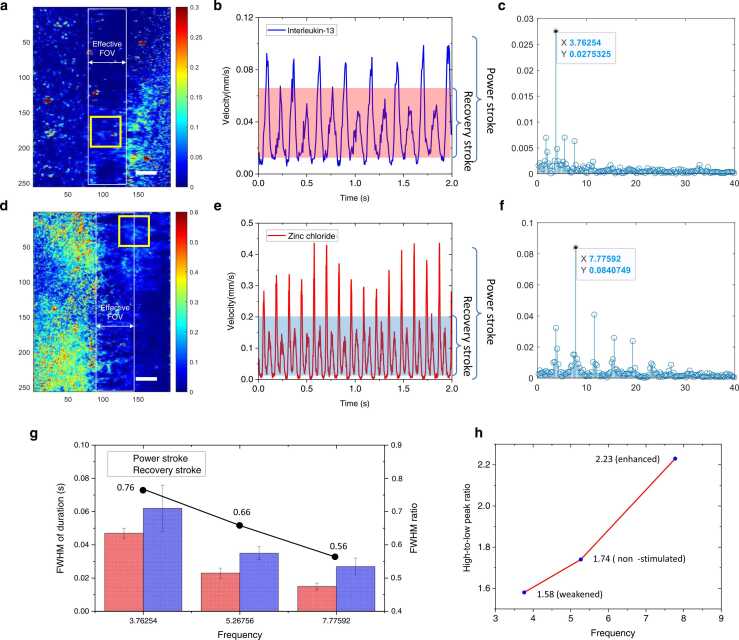
Beating waveforms of cilia with different treatments. **(a)** Velocity maps of cilia treated by IL-13. The overall FOV is 34.1 µm × 44.5 µm. (**b**) Mean velocity waveform extracted from the yellow box in **(a)**. **(c)** Fourier-transform of velocity waveform of **(b)**. **(d)** Velocity maps of cilia treated by ZnCl_2_. **(e)** Mean velocity waveform extracted from the yellow box in **(d)**. **(f)** Fourier-transform of velocity waveform of **(e)**. Scale bars in **(a)** and **(d)**: 5 µm. **(g)** Duration ratio between the power stroke and recovery stroke. **(h)** High-low velocity peak ratio.

The ciliary function may be altered by a variety of drugs and other substances, many of which alter the ciliary beat frequency. We studied ciliary activities in response to IL-13 and ZnCl_2_, which were used to reduce and increase cilia beating. Fig. 4**(a)** and 4**(d)** show the time-averaged velocity maps, which were obtained from the weakened cilia and enhanced cilia, respectively. The mean velocity waveforms, which were spatially averaged over areas marked by yellow boxes, were correspondingly plotted in Fig. 4**(b)**, and Fig. 4**(e)**. The beating period of weakened cilia was estimated 0.259 ± 0.019 s, and the beating period of enhanced was 0.129 ± 0.002 s. The beating frequencies found with Fourier analysis were 3.76 Hz (Fig. 4**(c)**) and 7.78 Hz (Fig. 4**(f)**) for weakened and enhanced cilia, respectively. The frequencies reported here were relatively low, and so was the non-stimulated frequency in Section 3.3. This was because the experiments were conducted at a room temperature of 22 ℃, significantly lower than the body temperature of 37 ℃ at which the maximum activity of the cells could be expected.

As shown in Fig. 4**(g)**, the FWHM of weakened power stroke duration was 0.047 ± 0.003 s, and the recovery stroke was 0.062 ± 0.014 s. The FWHM of enhanced power stroke duration was 0.015 ± 0.002 s, and the recovery stroke was 0.027 ± 0.005 s. The duration ratios between the power and recovery strokes were 0.76, 0.66, and 0.55, respectively, for the weakened, non-stimulated, and enhanced cilia. As shown in Fig. 4**(h)**, the high-to-low peak ratio of the weakened, non-stimulated, and enhanced cilia waveform were 1.58, 1.74, and 2.23, respectively. We found high-frequency ciliary beat was prone to sweep foreign objects in the airway in one direction. All of the evaluated parameters suggested that LSH-LSI could provide quantitative measures beyond the CBF changes to characterize the repones of motile cilia to different stimulations.

### 3.5 Spatial pattern of beating cilia

Besides the CBF, the CBP is a composite parameter that further characterizes the health and function of cilia. The temporal waveform characteristics, such as the peak values, high-to-low peak ratio, stroke durations, and duration ratio, could be potentially developed into quantitative CBP indicators. In principle, we could also take advantage of the rich information in the spatial patterns of cilia velocity distributions, which is the focus of this section.

**Fig. 5:**
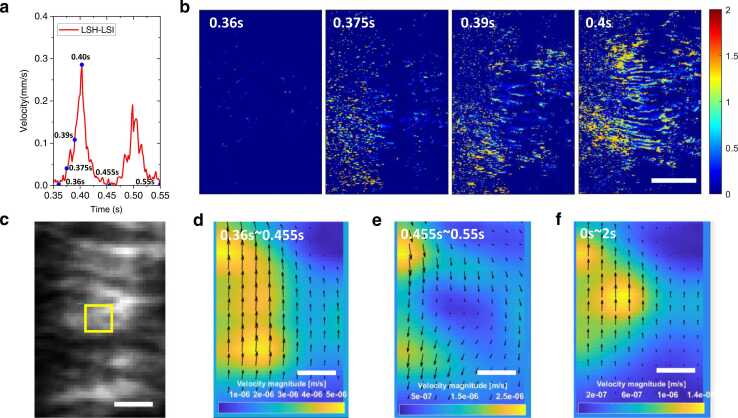
Ciliary beat spatial pattern. **(a)** One complete period of velocity waveform in Fig. 3**(e)**. **(b)** Ciliary beat velocity map at different time points. The unit of the color bar is mm/s. The FOV is 28.6 µm × 43.1 µm. Scalar bar: 10 µm. **(c)** Raw speckle image for PIV analysis. The FOV is the same as **(b)**. The size of the interrogation area (yellow box) is 8 × 8 pixels. **(d)** The ensemble PIV velocity map in 0.36 s∼0.455 s (power stroke). **(d)** The ensemble PIV velocity map in 0.455 s∼0.55 s (recovery stroke). **(f)** The ensemble PIV velocity map over 2 s. Scale bars in **(c)**∼**(f)**: 2 µm.

The velocity waveform in the yellow area in Fig. 3**(e)** was reproduced in Fig. 5**(a)**, which included one complete ciliary beat period. The power stroke was from around 0.36–0.455 s, and the recovery stroke was from around 0.455–0.55 s. The instantaneous scalar velocity maps at specific time points (0.36 s, 0.375 s, 0.39 s, and 0.40 s) were displayed in Fig. 5**(b)**, from which one could identify spatially connected patterns that were varying temporally. The complete scalar velocity map stack was rendered as a video (**Supplementary Video 1**), in which one could see wave-like behavior.

Supplementary material related to this article can be found online at doi:10.1016/j.csbj.2023.02.036.

The following is the Supplementary material related to this article Video S1..

Video S1

To create vector flow maps with directional information, raw laser speckles (Fig. 5**(c)**) were traced by using PIV [45]. In the PIVlab analysis tool, the interrogation area (yellow box) was set by 8 × 8 pixels, and the step size was 4 pixels. The images were processed with the ensemble correlation method. As shown in Fig. 5**(d),** the cilia moving direction during the power stroke was from the bottom to the top. In contrast, the direction of the recovery stroke was reversed (Fig. 5**(e)**). Fig. 5**(f)** is the PIV ensemble map evaluated over the 2 s period. It showed a net upward movement consistent with the power stroke direction. This could be used to quantify the efficiency for the particles or mucus in the airway being swept away in a preferred direction.

PIV analysis for a larger FOV was also performed. The results were combined to in another video (**Supplementary Video 2**) that revealed the spatiotemporal changes in cilia moving direction and velocity. However, long range correlations between different cilia clusters could not be readily identified. Further investigation is warranted to improve our experimental design.

Supplementary material related to this article can be found online at doi:10.1016/j.csbj.2023.02.036.

The following is the Supplementary material related to this article Video S2..

Video S2

## 4 Discussion

In this paper, we present the inverted light-sheet laser speckle imaging system and its application in cilia motion characterization. LSH-LSI is a label-free, functional, and quantitative imaging technique. The measured local velocities, as well as their temporal and spatial distributions, are absolute instead of relative. Therefore, it is possible to derive a wide range of indicators including the ciliary beat velocity amplitude, high-to-low peak ratio, and local moving directions that can potentially be used to evaluate the cilia function. Another important advantage of LSH-LSI is its optical sectioning capability. In fact, optical sectioning helps improve the quantification accuracy, as suggested by the comparison between LSH-LSI and SI-LSI in Section 3.2. However, this feature has not been fully exploited in our current study. We are interested in conducting further investigations in the future to explore multi-dimensional LSH-LSI imaging of motile cilia.

The advantages of oblique light-sheet laser speckle imaging system came at a price. With a large oblique angle (e.g., 45^o^), the effect FOV would be compromised by the limited DOF of the detection objective. In practice, the DOT could be extended by reducing the effective NA of the detection objective, leading to a lower spatial resolution. In our experiments, the effective FOV was 10–25 µm in length, much shorter than the effective length of the illumination light sheet (above 50 µm). The optical design of our setup needs to be further optimized to enjoy a larger FOV without compromising other performance indicators.

Due to light scattering, the LSH-LSI spatial resolution was inadequate to resolve individual cilia. As a result, it was difficult to directly characterize their geometrical changes during the beating process. However, our platform allowed the integration of a wide-field system which was a bright-field microscope in the current configuration but could also be configured as a DIC microscope. The combined system would enable a more comprehensive and complementary evaluation of cilia motion with multiple modalities. For example, we performed an autocorrelation analysis of the transmission image stack, enclosed in the white box in Fig. 3**(a)**. Individual cilia cells could be identified in the processed images. The processed images were further combined to create as a movie (**Supplementary Video 3**), which showed metachronal waves usually occur during the transfer of mucus or particles.

Supplementary material related to this article can be found online at doi:10.1016/j.csbj.2023.02.036.

The following is the Supplementary material related to this article Video S3..

Video S3

The spatiotemporal changes in cilia moving velocity and direction carry rich functional information. Many approaches have been proposed in the past ten years to capture the vector velocity maps. Among them are microbeads labelling based methods [46], [47], [48] and ink labelling based methods [44], [49], [50], which have been successfully developed by different research groups. However, a label-free technique is always desirable if it could provide similar information or even more.

In conclusion, we have built a novel light-sheet laser speckle imaging system, which is coupled with wide-field light microscope for comprehensive cilia motion evaluation. The feasibility of using the LSH-LSI technique to map the CBF and CBP has been validated across different conditions. Light sheet speckle imaging, as a quantitative imaging method, is able to accurately measure the cilia beat frequency and differentiate between various beat patterns with non-conventional indicators. Our present study establishes a foundation for the continuing development of an in vitro cilia imaging platform that may aid in the investigation of a variety of cilia-related airway diseases.

## CRediT authorship contribution statement

N. C. and D. W. conceived the initial idea and supervised this work. K. L. built the optical system. K. L. and J. L. performed imaging experiments. M. T. and J. L. provided and prepared the imaging samples. K. L. analyzed the imaging data. S. S. assisted in optical design and system optimization. K. L. and J. L. wrote the manuscript. All authors contributed to the discussion of the manuscript.

## Competing financial interests

The authors declare no competing financial interests.
